# Network of Interactions between the Mut Domains of the E2 Protein of Atypical Porcine Pestivirus and Host Proteins

**DOI:** 10.3390/genes15080991

**Published:** 2024-07-27

**Authors:** Yuai Yang, Guangfei Jiang, Weiqi He, Xin Tian, Huanli Zheng, Bin Xiang, Yongke Sun

**Affiliations:** 1 College of Veterinary Medicine, Yunnan Agricultural University, Kunming 650201, China; yangyuai2008@126.com (Y.Y.); 18887036121@163.com (G.J.); hwq727@126.com (W.H.); 18787746974@163.com (X.T.); 2021060@ynau.edu.cn (B.X.); 2 Yunnan Animal Health Supervision Institute, Kunming 650201, China; zhh3442536@126.com

**Keywords:** APPV, protein interaction, Mut domains, E2 protein, SSR4

## Abstract

Atypical porcine pestivirus (APPV) can cause congenital tremor type A-II in neonatal piglets, posing a significant threat to swine herd health globally. Our previous study demonstrated that the Mut domains, comprising 112 amino acids at the N-terminus, are the primary functional regions of the E2 protein of APPV. This study identified 14 host cellular proteins that exhibit potential interactions with the Mut domains of the E2 protein using yeast two-hybrid screening. Using bioinformatics analysis, we discovered that the Mut domains of the E2 protein might exert regulatory effects on apoptosis by modulating energy metabolism within the mitochondria. We also conducted co-immunoprecipitation, glutathione S-transferase pull-down, and immunofluorescence assays to confirm the interaction between the Mut domains of the E2 protein and cathepsin H and signal sequence receptor subunit 4 (SSR4). Ultimately, SSR4 enhanced APPV replication in vitro. In summary, our study successfully elucidated the interactions between the Mut domains of the E2 protein and host cell protein, predicted the potential pathways implicated in these interactions, and demonstrated SSR4 involvement in APPV infection. These significant findings contribute valuable knowledge toward a deeper understanding of APPV pathogenesis and the role of the Mut domains of the E2 protein in this intricate process.

## 1. Introduction

Congenital tremor (CT) is a neurological disease in newborn piglets characterized by tremors and ataxia that severely affects the pig industry. Based on histopathological lesions in the central nervous system, CTs are classified into the following six types: types AI–V and B [1]. In 2015, atypical porcine pestivirus (APPV) was first identified in porcine serum samples from the United States using metagenomic sequencing [2]. During the same period, APPV was also detected in the serum of asymptomatic and apparently healthy adult pigs in Germany [3]. Notably, the detection of APPV in pig serum samples from Spain in 1997 [4] and Switzerland in 1986 [5] suggests a prolonged circulation of the virus within pig herds. In 2016, two studies showed that APPV was associated with CT type A-II in newborn piglets by inoculating pregnant sows with tissues from APPV-infected pigs [6,7]. APPV has also been reported in swine herds in Sweden [8], the Netherlands [7], Austria [9], China [10], Brazil [11], Hungary [12], Japan [13], and Canada [14]. It has also been detected in wild boars in South Korea [15], Italy [16], Spain [17], Germany [18], and Sweden [19], with prevalence rates of 0.78% (15/2297), 0.69% (3/430), 0.23% (1/437), 19.08% (87/456), and 72% (433/595), respectively. Since APPV was first confirmed in swine herds in Guangdong province in China in 2016 [10], it has been reported in Guangxi, Yunnan, Guizhou, Anhui, Fujian, Jiangsu, Heilongjiang, Liaoning, Chongqing, Sichuan, Jiangxi, Henan, Shandong, and Hubei provinces [20,21]. Consequently, APPV poses a significant threat to the global swine industry.

After APPV infection, piglets can show neurological symptoms, such as tremors, ataxia, and impaired ability to stand and walk, while adult pigs typically do not show any clinical signs [9]. The severity of the disease can vary, with some piglets exhibiting only mild symptoms and others succumbing shortly after birth. The mortality rate of CT piglets associated with APPV was 60% in commercial pigs on a Chinese pig farm [22], 24.6% on a Canadian pig farm [14], and 20% on a Brazilian pig farm [11]. Previous studies have shown that APPV genomes can be detected in semen, preputial swabs, preputial fluids, the central nervous system, and lymphoid tissues [23,24]. Horizontal and transplacental transmissions have also been observed [6,7,25].

APPV was classified as Pestivirus K, which is an enveloped, highly variable RNA virus with a genome of about 12.3 kb belonging to the family Flaviviridae. This RNA encodes four structural proteins (C, E^rns^, E1, and E2) and eight non-structural proteins (N^pro^, P7, NS2, NS3, NS4A, NS4B, NS5A, and NS5B) [26]. Genome analysis revealed that APPV exhibits relatively low similarity (25–28%) to well-known pestiviruses, such as classical swine fever virus (CSFV), bovine viral diarrhea virus, and border disease virus. Furthermore, significant genetic diversity has been observed among different APPV strains, leading to their classification into at least four distinct groups [20]. NS3 is a multifunctional viral protein with protease, helicase, and nucleoside 5′-triphosphatase activities [27]. The N^pro^ of APPV has also been found to impede interferon-β production through its interference with the phosphorylation of interferon regulatory factor 3 [28]. Moreover, the E2 protein, a major component of APPV, can induce a Th2-type immune response in mice, indicating that the E2 protein could be developed as an effective subunit vaccine against APPV infection [29].

In our previous investigations, we provided evidence for the significance of the Mut domains, comprising 112 amino acids located at the N-terminus of the viral structural protein E2 as a crucial virulence domain for APPV [30]. Previous studies have established the pivotal role of interactions between viral and cellular proteins in the replication and virulence of viruses [31,32]. The yeast two-hybrid (Y2H) system has demonstrated efficacy in analyzing protein interactions [33,34]. Therefore, to identify potential host proteins that may interact with the Mut domains of the E2 protein, a cDNA library was created from immortalized swine umbilical vein endothelial cells (SUVECs). The Y2H system was used to identify cellular proteins that interact with the Mut domains of the E2 protein. These interactions were subsequently validated by immunofluorescence, co-immunoprecipitation, and glutathione S-transferase (GST) pull-down assays. Protein interaction network, Gene Ontology (GO) enrichment, and Kyoto Encyclopedia of Genes and Genomes (KEGG) pathway analyses were performed to map the interactions among these proteins. The interacting protein, signal sequence receptor subunit 4 (SSR4), a protein that regulates the retention of endoplasmic reticulum (ER) resident proteins, exhibited the ability to enhance APPV replication in vitro.

## 2. Materials and Methods

### 2.1. Cells, Viruses, Antibodies, and Reagents

SUVECs were kindly provided by Professor Yanming Zhang of the College of Veterinary Medicine, Northwest A&F University. Human embryonic kidney 293T (HEK-293T; CRL-3216, ATCC) and porcine kidney (PK) 15 (CCL-33, ATCC) cells were obtained from the American Type Culture Collection. These cell lines were cultured in Dulbecco’s Modified Eagle’s Medium (Gibco, Waltham, MA, USA) with 10% fetal bovine serum at 37 °C in a 5% CO_2_ atmosphere. HEK-293T cells are commonly selected for co-immunoprecipitation assays, co-localization analysis, and expression of exogenous proteins used in GST pull-down assays because of their high transfection efficiency, as described previously [34]. APPV (APPV-YN) was isolated from Yunnan Province and stored in our laboratory at Yunnan Agricultural University. Rabbit anti-FLAG, anti-GFP, anti-MYC, anti-GST, anti-tubulin, anti-glyceraldehyde-3-phosphate-dehydrogenase (GAPDH), and anti-beta(β)-actin monoclonal antibodies were obtained from Beyotime (Hefei, China). The rabbit anti-SSR4 and anti-APPV NS5 polyclonal antibodies were purchased from ABclonal (Wuhan, China) and BIOESN (Shanghai, China), respectively. Furthermore, the secondary antibodies included Cy3-conjugated goat anti-mouse and anti-rabbit immunoglobulin G (IgG) (H+L) (Beyotime).

### 2.2. Construction of Recombinant Expression Plasmids

To construct the recombinant expression plasmids pGBKT7-Mut, pEGFP-Mut, and pET-CST-Mut, the Mut region (336 bp) of the E2 gene of APPV was amplified from the genomic RNA of the APPV-YN strain and subsequently cloned into pGBKT7, pEGFP, and pET-GST vectors. The porcine genes cathepsin H (*CTSH*) and *SSR4* were amplified by PCR using cDNA from PK15 cells as a template and subsequently cloned into pCMV-tag4A with a C-terminal FLAG tag to obtain pCMV-tag4A-CTSH and pCMV-tag4A-SSR4, respectively. Table 1 presents the primers used in this study. All recombinant expression plasmids were further verified by sequencing.

### 2.3. Y2H Screening

Firstly, the SUVEC Y2H cDNA library was constructed. Then, we confirmed that the bait pGBKT7-mut had no self-activating activity or toxic effects on yeast. Next, pGBKT7-mut was transformed into the Y2H Gold strain and screened against the SUVEC cDNA library, as described previously [33].

### 2.4. Functional Analysis of Interacting Proteins

The 14 potential porcine binding targets of the Mut region of the E2 gene of APPV identified by the Y2H assay were input into the STRING database (https://string-db.org/, accessed on 1 November 2023), and host hits that may directly or indirectly interact with these targets were obtained. Mut–cellular protein interaction networks were annotated and visualized using Cytoscape (version 3.10.1).

GO and KEGG enrichment analyses and the visualization of these identified host proteins interacting with the Mut domains of the E2 protein were performed using KOBAS 3.0 (http://bioinfo.org/kobas/, accessed on 1 November 2023).

### 2.5. Glutathione S-Transferase Pull-Down Assays

Recombinant GST or GST-Mut protein was synthesized in *Escherichia coli* (*E. coli*) BL21 and subsequently purified using BeyoGold GST-tag purification resin. After purification, the proteins were incubated with 300 μL of lysates derived from the cells transfected with pCMV-tag4A-CTSH, pCMV-tag4A-SSR4, or pCMV-tag4A vector for 2 h at 4 °C. Subsequently, the proteins were subjected to a Western blotting (WB) assay after five washes.

### 2.6. Co-Localization Analysis in Cells

pEGFP-Mut plasmid was co-transfected with pCMV-tag4A-CTSH or pCMV-tag4A-SSR4 into HEK-293T cells. Subsequently, an indirect immunofluorescence assay was conducted using rabbit anti-FLAG monoclonal antibody and Cy3 goat anti-rabbit IgG (H+L). Nuclei were stained with 4′,6-diamidino-2-phenylindole. The cells were observed and recorded using a fluorescence microscope (Shunyu, Ningbo, China).

### 2.7. Co-Immunoprecipitation Assays

pEGFP-Mut plasmid was co-transfected with pCMV-tag4A-CTSH or pCMV-tag4A-SSR4 into HEK-293T cells. After 24 transfections, the cells were lysed using cell lysis buffer (Beyotime) for WB and immunoprecipitation assays. The resulting cell lysate was incubated overnight with the anti-GFP rabbit antibody and subsequently incubated with protein A/G agarose gels for 2 h. The immunoprecipitation complex was subjected to five washes with Tris Buffered Saline Tween-20, mixed with loading buffer, and subsequently used for WB analysis. Overall, three independent experiments were conducted.

### 2.8. The Effects of Signal Sequence Receptor Subunit 4 on APPV Infection

Small interfering RNA (siRNA) sequences targeting the *SSR4* gene were designed and synthesized by Sangon Company (Shanghai, China). The overexpression plasmid, pCMV-tag4A-SSR4, was prepared as described above. PK15 cells were transfected with the overexpression plasmid or siRNA using Lipofectamine 2000 (Invitrogen, Carlsbad, CA, USA) according to the manufacturer’s protocol. siRNA knockdown efficiency was determined by quantitative real-time PCR (qRT-PCR) and Western blot analysis. Next, the cells were inoculated with the APPV strain, APPV-YN, at a multiplicity of infection (MOI) of 0.1 after 24 h transfection. Cell lysates were collected at 24 and 48 h post-infection and stored for subsequent analysis.

### 2.9. Western Blotting Assay

Cells were lysed using a radioimmunoprecipitation assay buffer containing phenylmethanesulfonyl fluoride, and protein concentration was determined using a bicinchoninic acid Protein Assay Kit (Thermo Fisher Scientific, Waltham, MA, USA). Denatured proteins were separated using sodium dodecyl sulfate-polyacrylamide gel electrophoresis and transferred onto nitrocellulose membranes (Millipore, Billerica, MA, USA). After 1 h incubation at 37 °C in 5% skim milk to block non-specific binding, the membrane was exposed to primary antibodies overnight at 4 °C, followed by incubation with corresponding secondary antibodies for another 1 h at 37 °C. Finally, the membranes were visualized using a chemiluminescence imaging system and analyzed using ImageJ software (Version 1.53) for three independent experiments.

### 2.10. Quantitative Real-Time Polymerase Chain Reaction

Total RNA was collected from the PK15 cells after being transfected with siRNA using a RNeasy Mini kit (Qiagen, Valencia, CA, USA) and was subsequently reverse-transcribed into cDNA using ReverTra Ace qPCR RT Master Mix (Toyobo, Osaka, Japan). qRT-PCR was performed in three independent experiments, as previously described [35]. It was performed in a 7500 Fast Real-Time PCR system (Applied Biosystems, Rotkreuz, Switzerland) using the following cycle parameters: 1 cycle at 95 °C for 60 s followed by 40 cycles of 95 °C for 15 s and 60 °C for 35 s. Finally, SSR4 expression was normalized to a β-actin, and the relative expression level of SSR4 mRNA was determined using the 2^−∆∆CT^ method.

### 2.11. Statistical Analysis

The experiments were conducted in triplicate, and statistical analysis was performed using GraphPad Prism 9.5.1 (GraphPad Software Inc., Boston, MA, USA). Student’s *t*-test for pairwise comparisons or one-way ANOVA/Dunn’s multiple comparison test were used for multiple comparisons, and the results are expressed as means ± standard deviation. Statistical significance was determined at * *p* < 0.05 and ** *p* < 0.01.

## 3. Results

### 3.1. Screening of the Mut Domains of the E2 Protein of Atypical Porcine Pestivirus Interacting Proteins Using the Y2H System

The recombinant bait plasmid pGBKT7-Mut was screened against the SUVEC cDNA library. Fourteen blue colonies were collected from the quadruple dropout medium plates and tested by PCR for the library plasmids. Fourteen proteins (high identity > 99% proteins in NCBl) were identified as potential binding partners of the Mut domains of the E2 protein of APPV-YN. Table 2 presents their potential functions using information from the UniProt database.

### 3.2. Construction and Analysis of the Mut Domains of the E2 Protein–Cellular Protein Interaction Network

**Figure 1:**
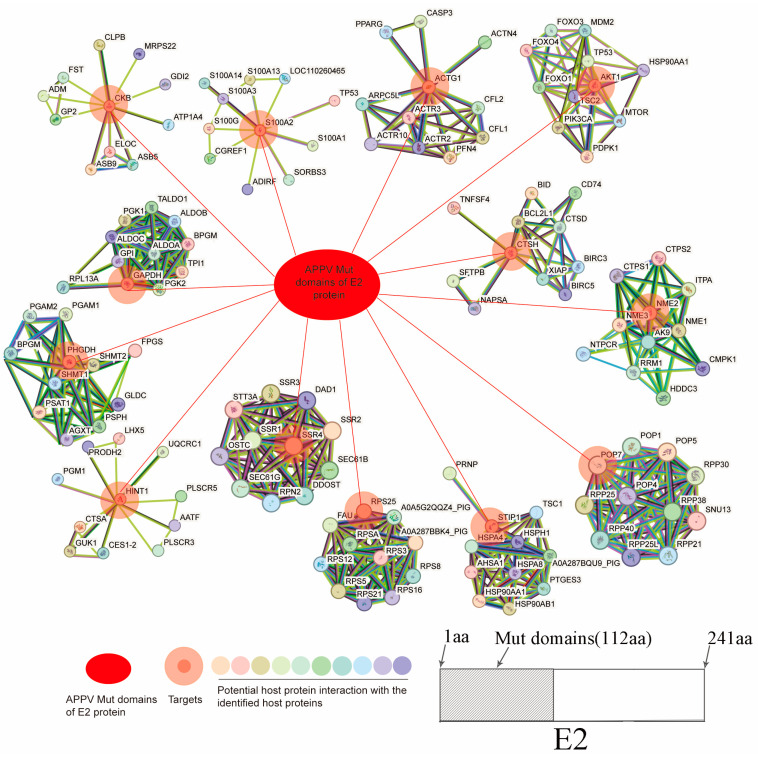
Network of cellular proteins. The network of host proteins interacting with the Mut domains of the E2 protein of APPV was constructed based on the STRING 9.0 database. APPV, atypical porcine pestivirus.

The potential porcine protein RPS26 was not logged in the STRING database (https://string-db.org/). Thus, the interaction network between the potential 13 porcine proteins (excluding RPS26) and Mut domains of the E2 protein of APPV was completed via STRING database analysis and Cytoscape. A total of 141 nodes representing APPV E2 and 140 host proteins were connected via 477 edges (Figure 1). The red ovals represent the Mut domains of the E2 protein of APPV, double circles signify the host proteins that interact with the Mut domains of the E2 protein, and other circles with different colors represent other host proteins that may interact with the 14 host proteins, among which nucleoside diphosphate kinase B, glyceralde-hyde-3-phosphate dehydrogenase (GAPDH), D-3-phosphoglycerate dehydrogenase, 40S ribosomal protein S25, small ribosomal subunit protein eS26, actin gamma 1, histidine triad nucleotide-binding protein 1, and RAC-alpha serine/threonine-protein kinase were identified as notable node proteins that exhibited interactions with each other. Conversely, CTSH, SSR4, ribonuclease P protein subunit p20, stress-induced phospho-protein 1, creatine kinase B-type, and S100 calcium-binding protein A2 were categorized as sub-notable node proteins because they only interacted with the Mut domains of the E2 protein. The detailed protein interaction information is shown in Supplementary Table S1.

### 3.3. GO and KEGG Enrichment Analyses

**Figure 2:**
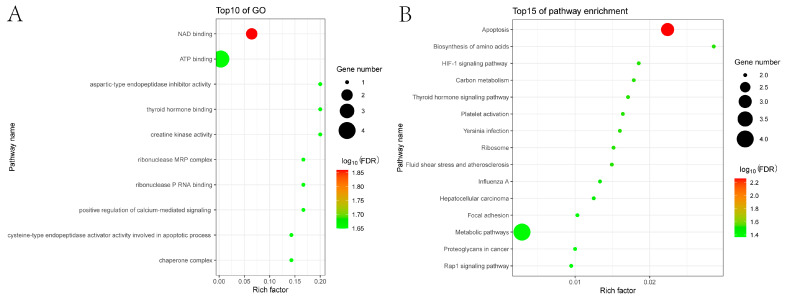
Enrichment analysis of the host proteins interacting with the Mut domains of the E2 protein of APPV using GO (**A**) and KEGG (**B**) databases. *p* < 0.05 was considered significant enrichment. GO, Gene Ontology; KEGG, Kyoto Encyclopedia of Genes and Genomes.

We conducted GO annotation and analysis to examine the roles and mechanisms of porcine proteins within the Mut domains of the APPV E2 protein–cellular protein interaction network. The results revealed that these host proteins were primarily associated with nicotinamide adenine dinucleotide (NAD) binding, ATP binding, aspartic-type endopeptidase inhibitor activity, thyroid hormone binding, creatine kinase activity, ribonuclease MRP complex, ribonuclease P RNA binding, and positive regulation of calcium-mediated signaling (Figure 2A).

Additionally, KEGG pathway enrichment analysis was performed to more comprehensively examine the cellular pathways of host proteins within the Mut domains of the APPV E2 protein–cellular protein interaction network. The results indicated that these host proteins were significantly enriched, primarily in apoptosis, amino acid biosynthesis, carbon metabolism, and metabolic pathways, which may respond by driving mitochondrial energy metabolism to influence APPV replication. Furthermore, various other pathways also showed enrichment for genes involved in the hypoxia-inducible factor 1 signaling pathway, thyroid hormone signaling pathway, influenza A, and Rap1 signaling pathway (Figure 2B).

### 3.4. Mut Domains of the E2 Protein Interact with CTSH and SSR4

**Figure 3:**
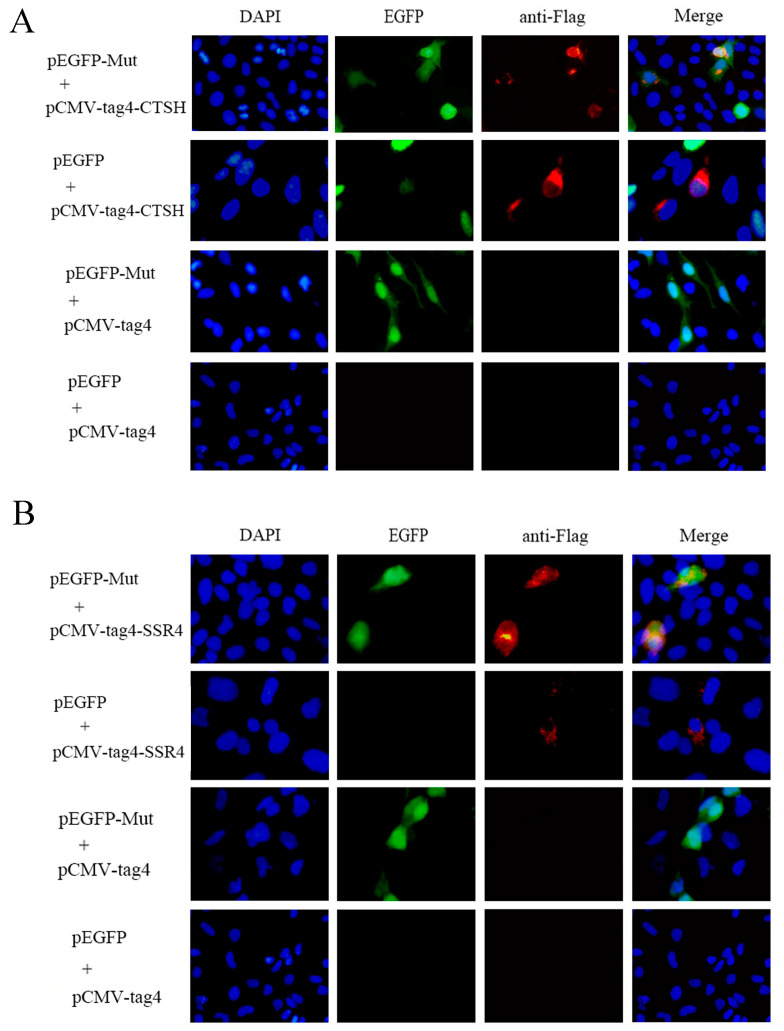
The Mut domains of the E2 protein co-localize with CTSH (**A**) and SSR4 (**B**) proteins in PK15 cells. To achieve this, plasmids expressing Flag-SSR4 or Flag-CTSH, along with plasmids expressing EGFP-Mut or EGFP, were co-transfected into the cells. After 48 h, the cells were fixed with 4% paraformaldehyde, stained with mouse anti-Flag (Red), and DAPI (Blue), and subsequently analyzed using a fluorescence microscope. Green indicates the EGFP-Mut or EGFP proteins. Yellow indicates colocalization of the Mut domains of the E2 protein co-localize with CTSH or SSR4 proteins in the overlay image.

**Figure 4:**
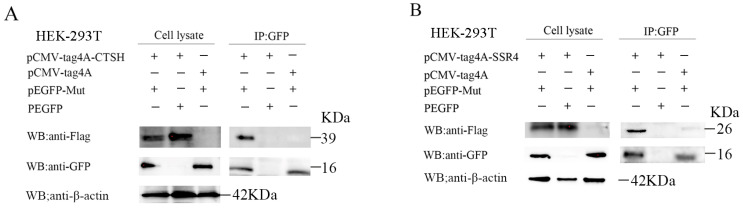
Interaction between the Mut domains of the E2 protein of APPV and CTSH (**A**) and SSR4 (**B**) proteins identified using a co-immunoprecipitation assay. HEK-293T cells were co-transfected with pEGFP-Mut and plasmids expressing Flag-SSR4 or Flag-CTSH. The cells were harvested after 24 h of transfection, and their lysates were subjected to immunoprecipitation using GFP antibodies, followed by immunoblotting with anti-Flag antibodies. HEK-293T, human embryonic kidney 293T; CTSH, cathepsin H; SSR4, signal sequence receptor subunit 4; APPV, atypical porcine pestivirus; GFP; green fluorescent protein.

**Figure 5:**
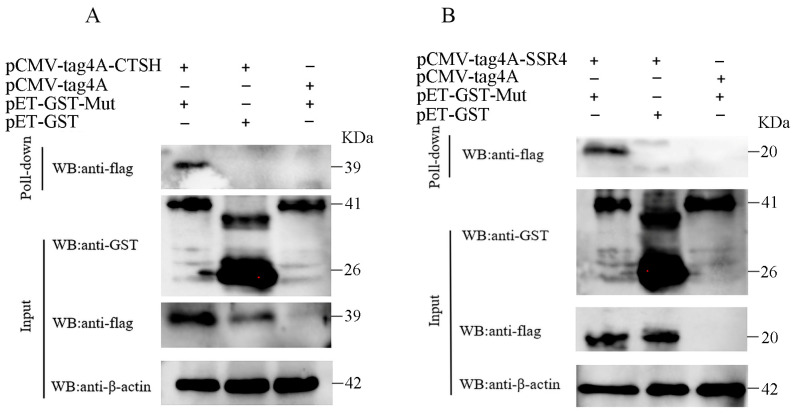
Interaction between the Mut domains of the E2 protein of APPV and CTSH (**A**) and SSR4 (**B**) proteins was identified using a GST pull-down assay. The GST-Mut recombinant proteins were obtained from prokaryotic cells, purified using GST beads, and subsequently incubated with Flag-SSR4 or Flag-CTSH expressed in HEK-293 T cells. After washing, the eluted complexes were analyzed using WB assay with anti-Flag antibodies. CTSH, cathepsin H; SSR4, signal sequence receptor subunit 4; APPV, atypical porcine pestivirus; WB, Western blotting; HEK-294T, human embryonic kidney 293T; GST, glutathione S-transferase.

Prior research has shown that CTSH and SSR4 are linked to apoptosis and ER stress, respectively, which are crucial for virus replication [36,37,38]. Moreover, CTSH was involved in the apoptotic process in the GO and KEGG analyses, and SSR4 was involved in the endomembrane system and endoplasmic reticulum in the GO analyses. To verify the interaction between the Mut domains of the E2 protein of APPV-YN and the identified porcine proteins using a Y2H system, immunofluorescence experiments were performed by co-transfecting pEGFP-Mut plasmids with pCMV-tag4A-CTSH or pCMV-tag4A-SSR4 plasmids into HEK-293T cells. The results showed that the Mut domains of the E2 protein co-localized with the CTSH and SSR4 proteins (Figure 3). Subsequently, a co-immunoprecipitation assay was conducted by co-transfecting pEGFP-Mut and pCMV-tag4A-CTSH or pCMV-tag4A-SSR4 into HEK-293T cells, and its results also provided additional evidence supporting the interaction between the Mut domains of the E2 protein and CTSH or SSR4 proteins (Figure 4). A GST pull-down assay was performed to validate this interaction. The Mut domains of the E2 protein tagged with CST protein were expressed in *E. coli*, while the CTSH or SSR4 proteins tagged with Flag were expressed in HEK-293T cells. The results demonstrate that GST-Mut successfully pulled down CTSH and SSR4 proteins (Figure 5). Overall, the Mut domains of the E2 protein of APPV could interact with porcine CTSH and SSR4 proteins.

### 3.5. Effects of SSR4 on the Replication of APPV

**Figure 6:**
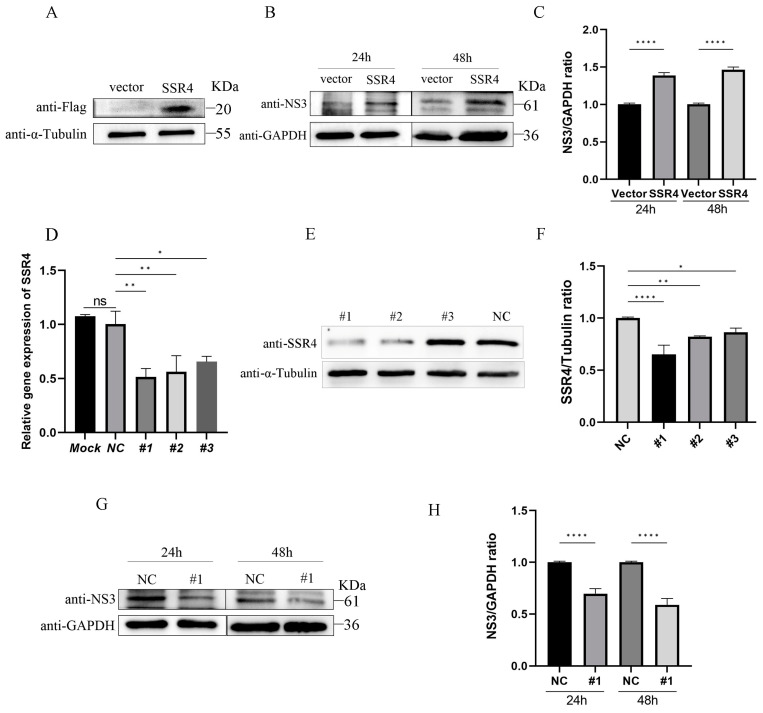
Effects of SSR4 on APPV replication. PK15 cells were transfected with pCMV-tag4A-SSR4 and pCMV-tag4A. After 24 h of transfection, the cells were infected with APPV at an MOI of 0.1. Subsequently, the cells were harvested at 24 and 48 hpi. The expression of exogenous SSR4 (**A**) and NS5 (**B**) of APPV was assessed using WB assay. The chart (**C**) shows the quantification of NS3/GAPDH in (**B**). Additionally, PK15 cells were co-transfected with non-targeting control siRNA (NC) or SSR4-targeting siRNAs. The inhibition efficiency of these three siRNAs was determined using quantitative real-time polymerase chain reaction (qRT-PCR) (**D**) and WB assay (**E**). The chart (**F**) shows the quantification of SSP4/α-Tubulin in (**E**). Furthermore, PK15 cells were transfected with NC or #1, followed by infection with APPV at an MOI of 0.1 after 24 h of transfection. Next, the cells were harvested at 24 and 48 hpi. WB analysis was conducted to determine the expression level of NS5 protein of APPV (**G**). The analysis of SSR4 silencing on the expression level of NS5 protein of APPV (**H**). Data presented are from three independent experiments and presented as mean ± SD. * *p* < 0.05, ** *p* < 0.01 and **** *p* < 0.0001. SSR4, signal sequence receptor subunit 4; Western blotting, WB; APPV, atypical porcine pestivirus; MOI, multiplicity of infection; siRNA, small interfering RNA; SD, standard deviation; PK15, porcine kidney 15.

Previous studies have demonstrated that human SSR4 could inhibit the replication of influenza virus [39]. To investigate the function of the porcine SSR4 gene during APPV infection, the recombinant expression plasmids pCMV-tag4A-SSR4 or pCMV-tag4A were transfected into PK15 cells, and the cells were inoculated with APPV at an MOI of 0.1 after 24 h of transfection. The results showed that the overexpression of SSR4 significantly increased APPV replication at the protein level 24 and 48 hpi (Figure 6A–C). Subsequently, the SSR4-specific siRNA was transfected into PK15 cells, resulting in a notable decrease in endogenous SSR4 expression at the mRNA and protein levels (Figure 6D–F). Furthermore, the infected SSR4 knockdown cells exhibited reduced viral NS3 protein levels at 24 and 48 hpi compared to control cells (Figure 6G,H). These observations strongly suggest that the porcine SSR4 gene plays a crucial role in facilitating APPV replication.

## 4. Discussion

APPV has been frequently documented worldwide since its initial report in 2015 [40], posing a significant threat to the swine industry. Recent studies have shed light on the role of virus-host interactions in determining viral virulence [41,42]. These interactions encompass various stages of the viral life cycle, such as virus adsorption, internalization, replication, assembly, and release, all of which heavily rely on the interplay between viral and cellular proteins. The E2 protein is a prominent constituent of APPV, and our previous research indicated that Mut domains serve as the primary functional regions of the E2 protein [30]. Consequently, we successfully identified the host proteins that interact with the Mut domains of the E2 protein in this study.

Earlier investigations have shown that the E2 protein of CSFV, a widely recognized pestivirus, plays a role in virus adsorption, the induction of protective immunity, and swine virulence and could also interact with multiple host proteins, including dynactin subunit 6 [43], Torsin-1A [44], SERTA Domain Containing Protein 1 [45], Coiled-Coil Domain-Containing 115 [46], mitogen-activated protein kinase 2 [47], protein phosphatase 1 catalytic subunit β [48], annexin 2 [49], and thioredoxin 2 [50]. APPV is also classified as a member of the pestivirus genus, suggesting that its E2 protein plays a significant role in the viral infection cycle by interacting with host proteins [19]. In the present study, 14 host proteins that potentially interact with the Mut domains of the E2 protein were identified using the Y2H system. Subsequently, the interactions between the Mut domains of the E2 protein and CTSH and SSR4 proteins from swine were confirmed using co-immunoprecipitation, GST pull-down, and immunofluorescence assays. Based on the GO analysis, the identified host proteins may be linked to energy metabolism, specifically NAD and ATP binding. KEGG pathway enrichment analysis indicated a significant enrichment of apoptosis. Virus-induced apoptosis significantly affects viral pathogenesis [51]. CSFV exploits the apoptotic process to facilitate viral infection [52]. During the initial phase of infection, CSFV suppresses apoptosis through the non-structural proteins N^pro^ and NS2, thereby promoting viral replication [53,54]. However, CSFV initiates substantial apoptosis in the later stages of CSFV infection, resulting in extensive harm to host tissue cells and facilitating the dissemination of viral progeny [55,56]. The mitochondria are pivotal for energy metabolism and play a crucial role in apoptosis [57]. Consequently, we speculated that the Mut domains of the E2 protein of APPV modulate apoptosis by regulating energy metabolism within the mitochondria to facilitate viral replication.

The *SSR4* gene encodes the translocon-associated protein (TRAP) δ (TRAPδ), which is a subunit of the TRAP complex located in the ER. This complex primarily regulates protein transport across the ER membrane and N-linked glycosylation, and it has been suggested there is an association between the TRAP complex and ER stress [37]. In the present study, we observed that SSR4 promoted APPV replication in vitro. SSR4 was first reported to be involved in APPV infection. Previous studies have shown that ER stress plays a role in viral infection through autophagy, as observed in alpha herpesvirus [58], porcine reproductive and respiratory syndrome viruses [59], and porcine epidemic diarrheal viruses [60]. Therefore, the Mut domains of the E2 protein can plausibly interact with the TRAPδ protein, potentially regulating ER stress and ultimately influencing APPV infection. However, the molecular mechanism of the porcine SSR4 gene supporting APPV replication requires further investigation. We also identified an interaction between the Mut domains of the E2 protein and porcine CTSH, which was shown to regulate apoptosis [36]. Autophagy typically occurs before apoptosis and can prevent apoptosis. However, the role of CTSH and cross-talk between autophagy and apoptosis in APPV infection requires further investigation.

## 5. Conclusions

Our study showed that 14 host proteins, which interacted with the Mut domains of the E2 protein of APPV, were mainly involved in apoptosis, amino acid biosynthesis, carbon metabolism, and metabolic pathways. We also found that SSR4, which interacts with the Mut domains of the E2 protein, enhances APPV replication in vitro. Therefore, this study contributes to the existing understanding of E2-interacting proteins and sheds light on the mechanism by which the Mut domains of the E2 protein affect APPV infection.

## Figures and Tables

**Table 1:** Primers and small interfering RNA sequence information.

Name	Forward Primer (5′-3′)	Reverse Primers (5′-3′)
pGBKT7/pEGFP-Mut	GAATTCATGCTCACCACCACCTGG	GGATCCCCTATTGGGCAGACAAGA
pET-GST-Mut	GGATCCATGCTCACCACCTGG	GAATTCCCTATTGGGCAGACAAGA
pCMV-tag4A-CTSH	GAATTCATGTGGGCCGTCCTGTC	CTCGAGCACCAGGGGGATCGGG
pCMV-tag4A-SSR4	GAATTCATGGCGGCGCTGG	CTCGAGGGCCTGGATGTGGCTC
SSR4 (qRT-PCR)	ATCTCCACAGAGACCGTGTT	GCTGTAGGACTCCTCATCGAAG
β-actin (qRT-PCR)	CACCATTGGCAATGAGCGGTTC	AGGTCTTTGCGGATGTCCACGT
siSSR4-#1	GAUCACGCCCUCGUACUAUtt	AUAGUACGAGGGCGUGAUCtt
siSSR4-#2	GGAAGGCUCAGAGAAAUAAtt	UUAWUUCUCUGAGCCUUCCtt
siSSR4-#3	GCAAGAACAGGGUCCAGAACAtt	UGUUCUGGACCCUGUUCUUGCtt
Negative control (NC)	UUCUCCGAACGUGUCACGU	AAGAGGCUUGCACAGUGCA

**Table 2:** Potential binding partners of the Mut domains of the E2 protein of APPV.

Gene	Protein	NCBI Accession	Function
*SSR4*	Translocon-associated protein subunit delta	XM_003135482.5	Regulates the retention of ER resident proteins
*GAPDH*	Glyceraldehyde-3-phosphate dehydrogenase	XM_021091114.1	Modulates the organization and assembly of the cytoskeleton and innate immunity
*HINT1*	Histidine triad nucleotide-binding protein 1	XM_005661623.3	Involved in phosphorylation of ribonucleotide
*CTSH*	Cathepsin H	XM_017021951.2	Involved in proteolysis associated with protein catabolic process
*STIP1*	Stress-induced phosphoprotein 1	XM_003353794.4	Co-chaperone that binds to heat shock proteins 70 and 90
*POP7*	Ribonuclease P protein subunit p20	XM_013995472.2	Involved in rRNA processing, tRNA 5′-leader removal, and tRNA processing
*PHGDH*	D-3-phosphoglycerate dehydrogenase	XM_011541226.3	Involved in the early steps of L-serine synthesis in animal cells
*RPS25*	40S ribosomal protein S25	NM_001028.3	Component of the small ribosomal subunit
*CKB*	Creatine kinase B-type	XM_021081502.1	Reversibly catalyzes the transfer of phosphate between ATP and various phosphagens
*AKT1*	RAC-alpha serine/threonine-protein kinase	XM_021081500.1	Protein serine/threonine kinase activity
*NME2*	Nucleoside diphosphate kinase B	NM_001044610.2	Involved in the synthesis of nucleoside triphosphates other than ATP
*RPS26*	Small ribosomal subunit protein eS26	NM_001097481.2	Structural constituent of the ribosome
*S100A2*	S100 calcium-binding protein A2	XM_001929556.5	Calcium ion binding
*ACTG1*	Actin gamma 1	XM_003357928.4	Involved in various types of cell motility and maintenance of the cytoskeleton

ER, endoplasmic reticulum; ATP, adenosine triphosphate; APPV, atypical porcine pestivirus.

## Data Availability

The data presented in this study are available on request from the corresponding author due to their containing information that could compromise the privacy of research participants.

## Supplementary Materials

The following supporting information can be downloaded at: https://www.mdpi.com/article/10.3390/genes15080991/s1, File S1: Supplementary data file of uncropped blots; Table S1: The detailed protein interaction information.

## References

[1] Stenberg H., Jacobson M., Malmberg M. (2020). A review of congenital tremor type A-II in piglets. Anim. Health Res. Rev..

[2] Hause B.M., Collin E.A., Peddireddi L., Yuan F.F., Chen Z.H., Hesse R.A., Gauger P.C., Clement T., Fang Y., Anderson G. (2015). Discovery of a novel putative atypical porcine pestivirus in pigs in the USA. J. Gen. Virol..

[3] Postel A., Hansmann F., Baechlein C., Fischer N., Alawi M., Grundhoff A., Derking S., Tenhündfeld J., Pfankuche V.M., Herder V. (2016). Presence of atypical porcine pestivirus (APPV) genomes in newborn piglets correlates with congenital tremor. Sci. Rep..

[4] Muñoz-González S., Canturri A., Pérez-Simó M., Bohórquez J.A., Rosell R., Cabezón O., Segalés J., Domingo M., Ganges L. (2017). First report of the novel atypical porcine pestivirus in Spain and a retrospective study. Transbound. Emerg. Dis..

[5] Kaufmann C., Stalder H., Sidler X., Renzullo S., Gurtner C., Grahofer A., Schweizer M. (2019). Long-Term Circulation of Atypical Porcine Pestivirus (APPV) within Switzerland. Viruses.

[6] Arruda B.L., Arruda P.H., Magstadt D.R., Schwartz K.J., Dohlman T., Schleining J.A., Patterson A.R., Visek C.A., Victoria J.G. (2016). Identification of a Divergent Lineage Porcine Pestivirus in Nursing Piglets with Congenital Tremors and Reproduction of Disease following Experimental Inoculation. PLoS ONE.

[7] de Groof A., Deijs M., Guelen L., van Grinsven L., van Os-Galdos L., Vogels W., Derks C., Cruijsen T., Geurts V., Vrijenhoek M. (2016). Atypical Porcine Pestivirus: A Possible Cause of Congenital Tremor Type A-II in Newborn Piglets. Viruses.

[8] Stenberg H., Jacobson M., Malmberg M. (2020). Detection of atypical porcine pestivirus in Swedish piglets with congenital tremor type A-II. BMC Vet. Res..

[9] Schwarz L., Riedel C., Högler S., Sinn L.J., Voglmayr T., Wöchtl B., Dinhopl N., Rebel-Bauder B., Weissenböck H., Ladinig A. (2017). Congenital infection with atypical porcine pestivirus (APPV) is associated with disease and viral persistence. Vet. Res..

[10] Zhang K., Wu K., Liu J., Ge S., Xiao Y., Shang Y., Ning Z. (2017). Identification of atypical porcine pestivirus infection in swine herds in China. Transbound. Emerg. Dis..

[11] Gatto I.R.H., Harmon K., Bradner L., Silva P., Linhares D.C.L., Arruda P.H., de Oliveira L.G., Arruda B.L. (2018). Detection of atypical porcine pestivirus in Brazil in the central nervous system of suckling piglets with congenital tremor. Transbound. Emerg. Dis..

[12] Dénes L., Biksi I., Albert M., Szeredi L., Knapp D.G., Szilasi A., Bálint A., Balka G. (2018). Detection and phylogenetic characterization of atypical porcine pestivirus strains in Hungary. Transbound. Emerg. Dis..

[13] Kasahara-Kamiie M., Kagawa M., Shiokawa M., Sunaga F., Fukase Y., Aihara N., Shiga T., Kamiie J., Aoki H., Nagai M. (2022). Detection and genetic analysis of a novel atypical porcine pestivirus from piglets with congenital tremor in Japan. Transbound. Emerg. Dis..

[14] Dessureault F.G., Choinière M., Provost C., Gagnon C.A. (2018). First report of atypical porcine pestivirus in piglets with congenital tremor in Canada. Can. Vet. J. La Rev. Vet. Can..

[15] Choe S., Park G.N., Cha R.M., Hyun B.H., Park B.K., An D.J. (2020). Prevalence and Genetic Diversity of Atypical Porcine Pestivirus (APPV) Detected in South Korean Wild Boars. Viruses.

[16] Sozzi E., Salogni C., Lelli D., Barbieri I., Moreno A., Alborali G.L., Lavazza A. (2019). Molecular Survey and Phylogenetic Analysis of Atypical Porcine Pestivirus (APPV) Identified in Swine and Wild Boar from Northern Italy. Viruses.

[17] Colom-Cadena A., Ganges L., Muñoz-González S., Castillo-Contreras R., Bohórquez J.A., Rosell R., Segalés J., Marco I., Cabezon O. (2018). Atypical porcine pestivirus in wild boar (*Sus scrofa*), Spain. Vet. Rec..

[18] Cagatay G.N., Antos A., Meyer D., Maistrelli C., Keuling O., Becher P., Postel A. (2018). Frequent infection of wild boar with atypical porcine pestivirus (APPV). Transbound. Emerg. Dis..

[19] Stenberg H., Leveringhaus E., Malmsten A., Dalin A.M., Postel A., Malmberg M. (2022). Atypical porcine pestivirus-A widespread virus in the Swedish wild boar population. Transbound. Emerg. Dis..

[20] Ma H.L., Li W.T., Zhang M.J., Yang Z.X., Lin L.L., Ghonaim A.H., He Q.G. (2022). The Diversity and Spatiotemporally Evolutionary Dynamic of Atypical Porcine Pestivirus in China. Front. Microbiol..

[21] Sun X., Zhang Q., Shan H., Cao Z., Huang J. (2023). Genome characteristics of atypical porcine pestivirus from abortion cases in Shandong Province, China. Virol. J..

[22] Shen H., Liu X., Zhang P., Wang L., Liu Y., Zhang L., Liang P., Song C. (2018). Identification and characterization of atypical porcine pestivirus genomes in newborn piglets with congenital tremor in China. J. Vet. Sci..

[23] Liu J., Li Z., Ren X., Li H., Lu R., Zhang Y., Ning Z. (2019). Viral load and histological distribution of atypical porcine pestivirus in different tissues of naturally infected piglets. Arch. Virol..

[24] Yuan J., Han Z.Y., Li J., Huang Y.Z., Yang J.F., Ding H.X., Zhang J.Y., Zhu M.J., Zhang Y.Y., Liao J.D. (2017). Atypical Porcine Pestivirus as a Novel Type of Pestivirus in Pigs in China. Front. Microbiol..

[25] Sutton K.M., Lahmers K.K., Harris S.P., Wijesena H.R., Mote B.E., Kachman S.D., Borza T., Ciobanu D.C. (2019). Detection of atypical porcine pestivirus genome in newborn piglets affected by congenital tremor and high preweaning mortality1. J. Anim. Sci..

[26] King A.M.Q., Lefkowitz E.J., Mushegian A.R., Adams M.J., Dutilh B.E., Gorbalenya A.E., Harrach B., Harrison R.L., Junglen S., Knowles N.J. (2018). Changes to taxonomy and the International Code of Virus Classification and Nomenclature ratified by the International Committee on Taxonomy of Viruses (2018). Arch. Virol..

[27] Blome S., Beer M., Wernike K. (2017). New Leaves in the Growing Tree of Pestiviruses. Adv. Virus Res..

[28] Bashashati M., Chung D.H., Fallah Mehrabadi M.H., Lee D.H. (2021). Evolution of H9N2 avian influenza viruses in Iran, 2017–2019. Transbound Emerg. Dis..

[29] Zhang H.W., Wen W., Hao G.X., Chen H.C., Qian P., Li X.M. (2018). A Subunit Vaccine Based on E2 Protein of Atypical Porcine Pestivirus Induces Th2-type Immune Response in Mice. Viruses.

[30] Sun Y.-K., Kong L.-F., Zhang X.-M., Zheng H.-L., Lin M.-X., Chen H.-T., Yang Y.-A. (2012). Construction of a chimeric virus based on atypical classical swine fever virus Yunnan strain with Shimen strain. Chin. J. Prev. Vet. Med..

[31] Perrin-Cocon L., Diaz O., Jacquemin C., Barthel V., Ogire E., Ramière C., André P., Lotteau V., Vidalain P.O. (2020). The current landscape of coronavirus-host protein-protein interactions. J. Transl. Med..

[32] Wen S.B., Li X.T., Lv X.Y., Liu K., Ren J.Q., Zhai J.B., Song Y. (2023). Current progress on innate immune evasion mediated by N. protein of pestiviruses. Front. Immunol..

[33] Chen X.N., Chen X.J., Liang Y.F., Xu S.J., Weng Z.J., Gao Q., Huang Z., Zhang G.H., Gong L. (2022). Interaction network of African swine fever virus structural protein p30 with host proteins. Front. Microbiol..

[34] Fan J.D., Zhang M.R., Liu C.C., Zhu M.J., Zhang Z.L., Wu K.K., Li Z.Y., Li W.H., Fan S.Q., Ju C.M. (2020). The Network of Interactions Between Classical Swine Fever Virus Nonstructural Protein p7 and Host Proteins. Front. Microbiol..

[35] Xiang B., You R., Kang Y., Xie P., Zhu W., Sun M., Gao P., Li Y., Ren T. (2019). Host immune responses of pigeons infected with Newcastle disease viruses isolated from pigeons. Microb. Pathog..

[36] Chen Q., Qu S., Liang Z., Liu Y., Chen H., Ma S., Liu X. (2023). Cathepsin H Knockdown Reverses Radioresistance of Hepatocellular Carcinoma via Metabolic Switch Followed by Apoptosis. Int. J. Mol. Sci..

[37] Phoomak C., Cui W., Hayman T.J., Yu S.H., Zhao P., Wells L., Steet R., Contessa J.N. (2021). The translocon-associated protein (TRAP) complex regulates quality control of N-linked glycosylation during ER stress. Sci. Adv..

[38] Mehrbod P., Ande S.R., Alizadeh J., Rahimizadeh S., Shariati A., Malek H., Hashemi M., Glover K.K.M., Sher A.A., Coombs K.M. (2019). The roles of apoptosis, autophagy and unfolded protein response in arbovirus, influenza virus, and HIV infections. Virulence.

[39] Wang Q., Li J.P., Liang L.B., Zhao Y.H., Wen X., Kong F.D., Wang G.W., Shi W.J., Li Q.B., Jiang L. (2021). Interaction between influenza virus PA protein and host protein SSR4. Chin. J. Prev. Vet. Med..

[40] Pan S.N., Mou C.X., Chen Z.H. (2019). An emerging novel virus: Atypical porcine pestivirus (APPV). Rev. Med. Virol..

[41] Liang Y.Y. (2023). Pathogenicity and virulence of influenza. Virulence.

[42] Wang Z., Zhao J. (2019). Pathogenesis of Hypervirulent Fowl Adenovirus Serotype 4: The Contributions of Viral and Host Factors. Viruses-Basel.

[43] Borca M.V., Vuono E.A., Ramirez-Medina E., Azzinaro P., Berggren K.A., Singer M., Rai A., Pruitt S., Silva E.B., Velazquez-Salinas L. (2020). Structural Glycoprotein E2 of Classical Swine Fever Virus Interacts with Host Protein Dynactin Subunit 6 (DCTN6) during the Virus Infectious Cycle. J. Virol..

[44] Vuono E.A., Ramirez-Medina E., Velazquez-Salinas L., Berggren K., Rai A., Pruitt S., Espinoza N., Gladue D.P., Borca M.V. (2021). Structural Glycoprotein E2 of Classical Swine Fever Virus Critically Interacts with Host Protein Torsin-1A during the Virus Infectious Cycle. J. Virol..

[45] Vuono E.A., Ramirez-Medina E., Azzinaro P., Berggren K.A., Rai A., Pruitt S., Silva E., Velazquez-Salinas L., Borca M.V., Gladue D.P. (2020). SERTA Domain Containing Protein 1 (SERTAD1) Interacts with Classical Swine Fever Virus Structural Glycoprotein E2, Which Is Involved in Virus Virulence in Swine. Viruses.

[46] Vuono E.A., Ramirez-Medina E., Berggren K., Rai A., Pruitt S., Silva E., Velazquez-Salinas L., Gladue D.P., Borca M.V. (2020). Swine Host Protein Coiled-Coil Domain-Containing 115 (CCDC115) Interacts with Classical Swine Fever Virus Structural Glycoprotein E2 during Virus Replication. Viruses.

[47] Wang J.H., Chen S.C., Liao Y.J., Zhang E.Y., Feng S., Yu S.X., Li L.F., He W.R., Li Y.F., Luo Y.Z. (2016). Mitogen-Activated Protein Kinase Kinase 2, a Novel E2-Interacting Protein, Promotes the Growth of Classical Swine Fever Virus via Attenuation of the JAK-STAT Signaling Pathway. J. Virol..

[48] Vuono E.A., Ramirez-Medina E., Holinka L.G., Baker-Branstetter R., Borca M.V., Gladue D.P. (2019). Interaction of Structural Glycoprotein E2 of Classical Swine Fever Virus with Protein Phosphatase 1 Catalytic Subunit beta (PPP1CB). Viruses.

[49] Yang Z., Shi Z.X., Guo H.C., Qu H., Zhang Y., Tu C.C. (2015). Annexin 2 is a host protein binding to classical swine fever virus E2 glycoprotein and promoting viral growth in PK-15 cells. Virus Res..

[50] Li S., Wang J.H., He W.R., Feng S., Li Y.F., Wang X., Liao Y.J., Qin H.Y., Li L.F., Dong H. (2015). Thioredoxin 2 Is a Novel E2-Interacting Protein That Inhibits the Replication of Classical Swine Fever Virus. J. Virol..

[51] Carruthers V.B., Cotter P.A., Kumamoto C.A. (2007). Microbial pathogenesis: Mechanisms of infectious disease. Cell Host Microbe.

[52] Ma S.M., Mao Q., Yi L., Zhao M.Q., Chen J.D. (2019). Apoptosis, Autophagy, and Pyroptosis: Immune Escape Strategies for Persistent Infection and Pathogenesis of Classical Swine Fever Virus. Pathogens.

[53] Tang Q., Guo K., Kang K., Zhang Y., He L., Wang J. (2011). Classical swine fever virus NS2 protein promotes interleukin-8 expression and inhibits MG132-induced apoptosis. Virus Genes.

[54] Johns H.L., Doceul V., Everett H., Crooke H., Charleston B., Seago J. (2010). The classical swine fever virus N-terminal protease N(pro) binds to cellular HAX-1. J. Gen. Virol..

[55] Everett H., McFadden G. (1999). Apoptosis: An innate immune response to virus infection. Trends Microbiol..

[56] Gou H.C., Zhao M.Q., Fan S.Q., Yuan J., Liao J.D., He W.C., Xu H.L., Chen J.D. (2017). Autophagy induces apoptosis and death of T lymphocytes in the spleen of pigs infected with CSFV. Sci. Rep..

[57] Bock F.J., Tait S.W.G. (2020). Mitochondria as multifaceted regulators of cell death. Nat. Rev. Mol. Cell Biol..

[58] Ming S.L., Zhang S., Wang Q., Zeng L., Zhou L.Y., Wang M.D., Ma Y.X., Han L.Q., Zhong K., Zhu H.S. (2022). Inhibition of USP14 influences alphaherpesvirus proliferation by degrading viral VP16 protein via ER stress-triggered selective autophagy. Autophagy.

[59] Diao F.F., Jiang C.L., Sun Y.Y., Gao Y.N., Bai J., Nauwynck H., Wang X.W., Yang Y.Q., Jiang P., Liu X. (2023). Porcine reproductive and respiratory syndrome virus infection triggers autophagy via ER stress-induced calcium signaling to facilitate virus replication. PLoS Pathog..

[60] Zou D.H., Xu J.X., Duan X.L., Xu X., Li P.F., Cheng L.X., Zheng L., Li X.Z., Zhang Y.T., Wang X.H. (2019). Porcine epidemic diarrhea virus ORF3 protein causes endoplasmic reticulum stress to facilitate autophagy. Vet. Microbiol..

