# Supplementary material for: Network of Interactions between the Mut Domains of the E2 Protein of Atypical Porcine Pestivirus and Host Proteins
Source: Genes (Basel). 2024 Jul 27;15(8):991. doi: 10.3390/genes15080991 (PMC11354059; doi:10.3390/genes15080991)
Supplement: Supplementary file 1 [file genes-15-00991-s001.zip › File S1. supplementary data file of uncropped blots.pdf]

Figure 4-A-1

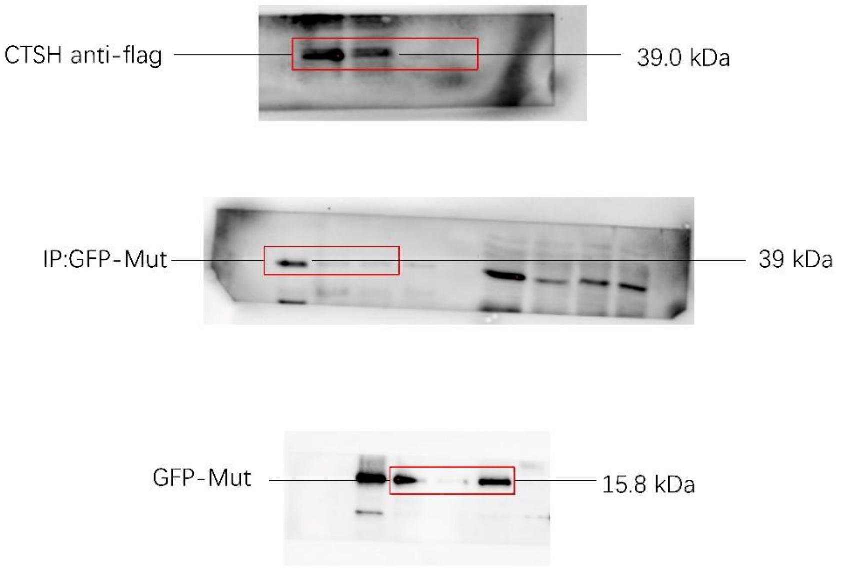

Figure 4-A-2

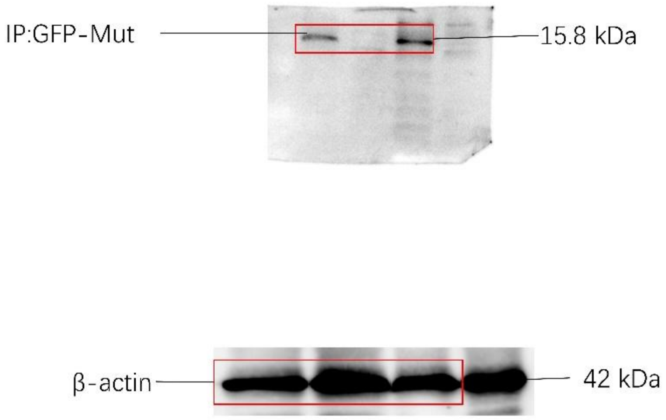

Figure 4-B-1

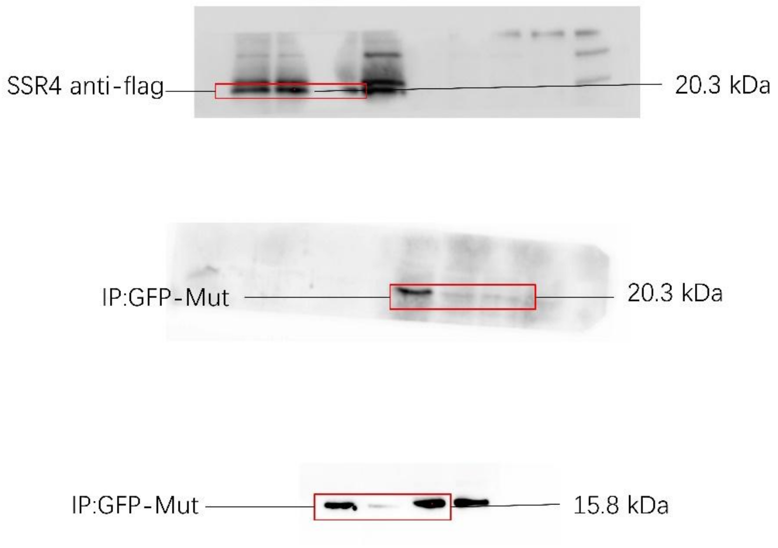

Figure 4-B-2

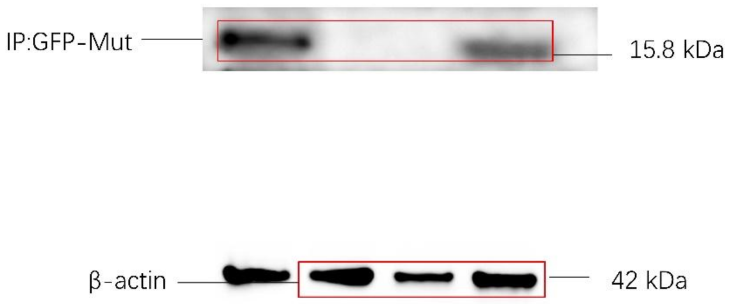

Figure 5-A-1

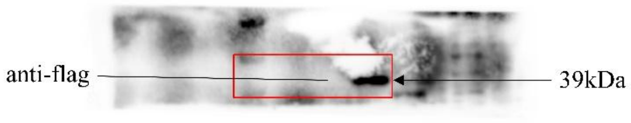

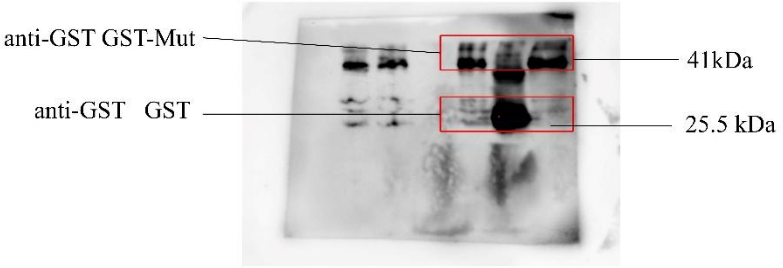

Figure 5-A-2

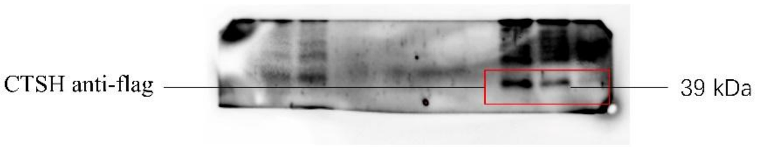

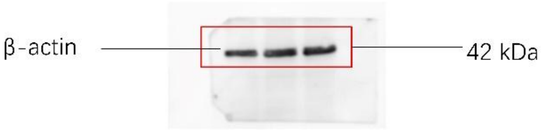

Figure 5-B-1

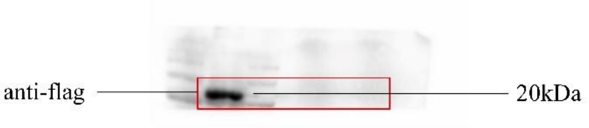

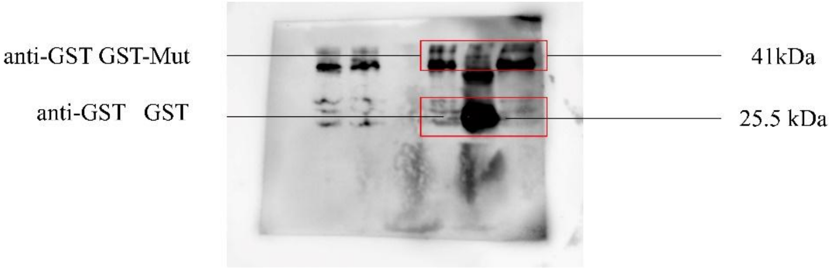

Figure 5-B-2

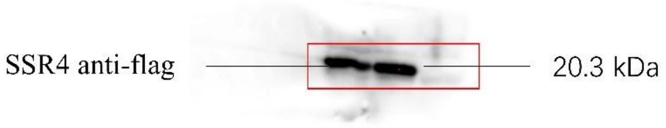

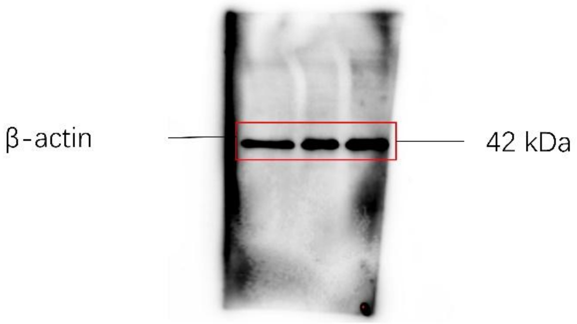

Figure6-A-1

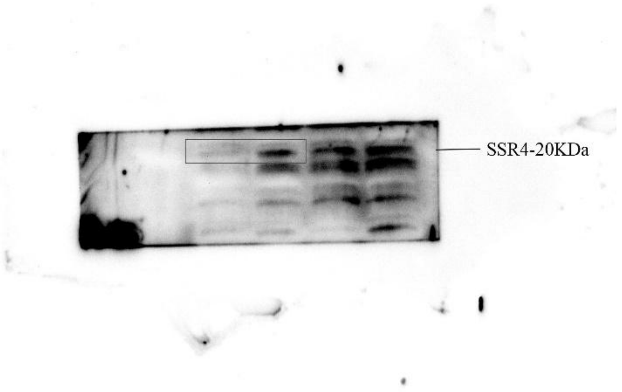

Figure6-A-2

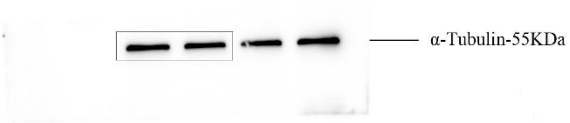

Figure6-B-1

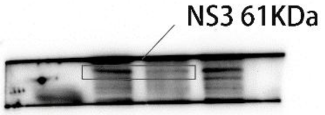

Figure6-B-2

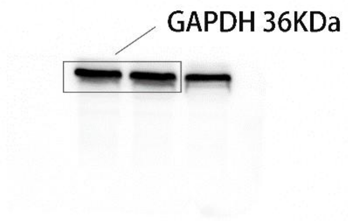

Figure6-B-3

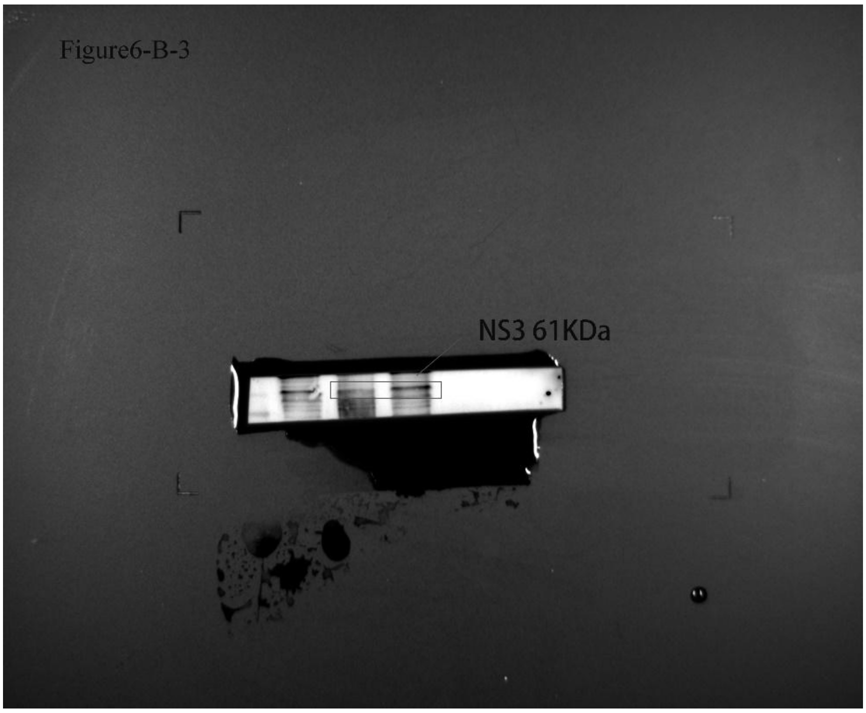

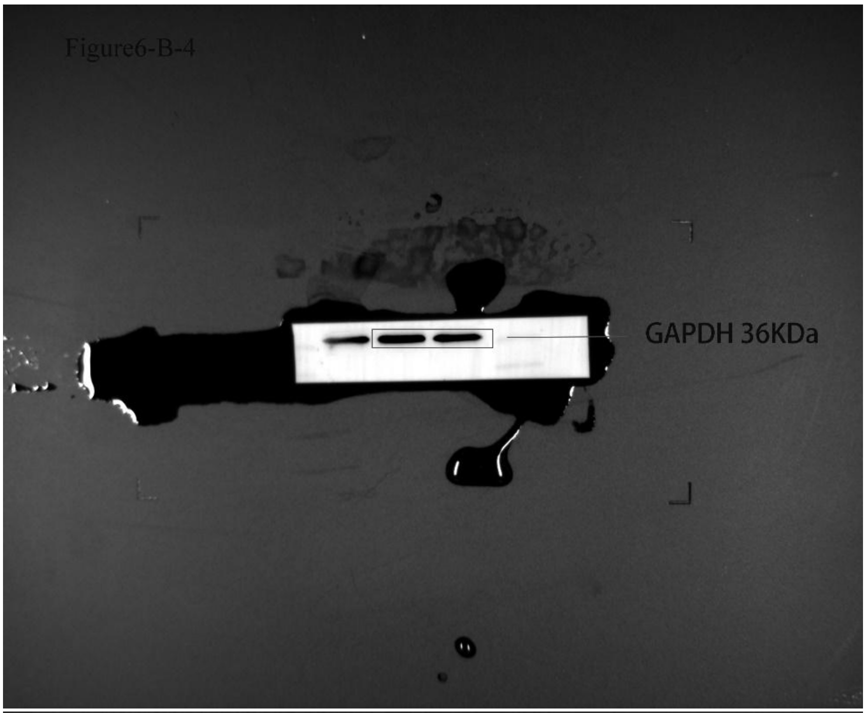
Figure6-B-4
GAPDH 36KDa

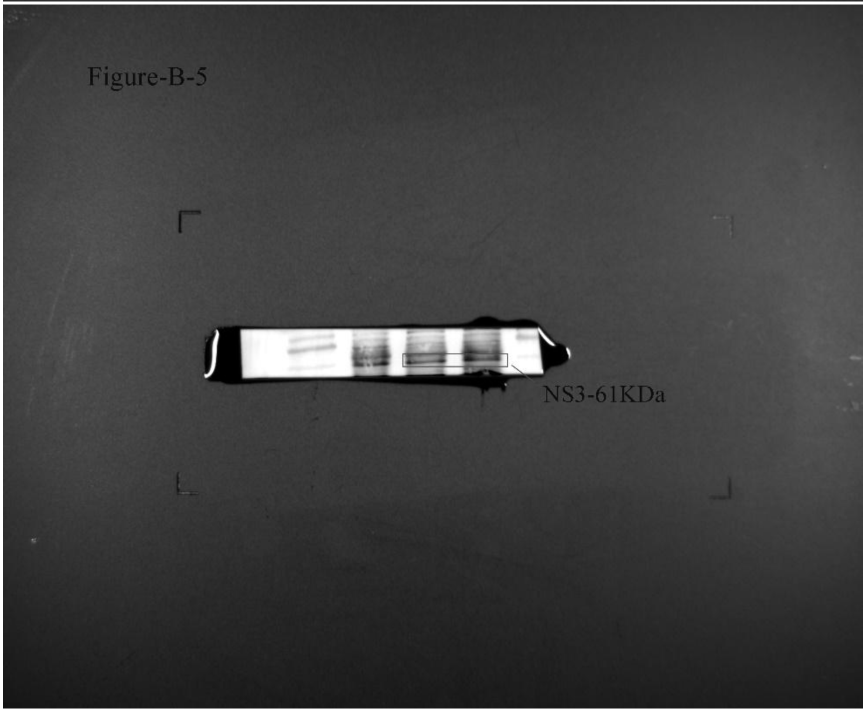
Figure-B-5
NS3-61KDa

Figure6-B-6

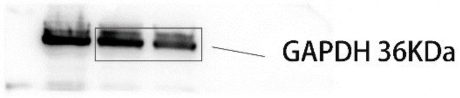

Figure6-B-7

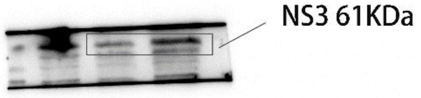

Figure6-B-8

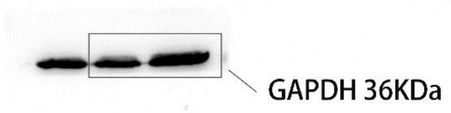

Figure6-B-9

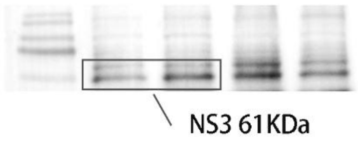

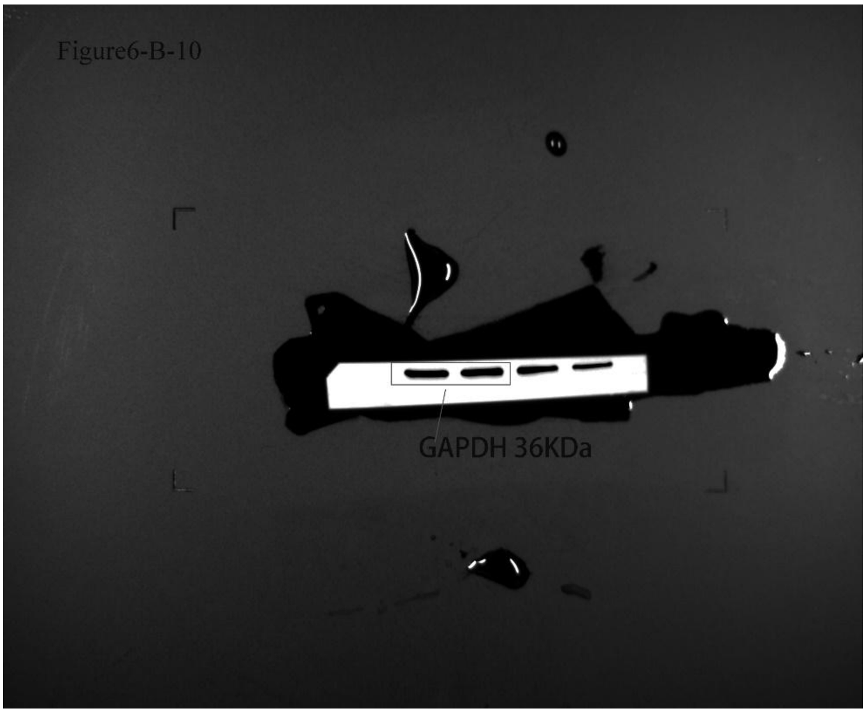

Figure6-B-11

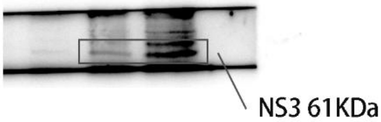

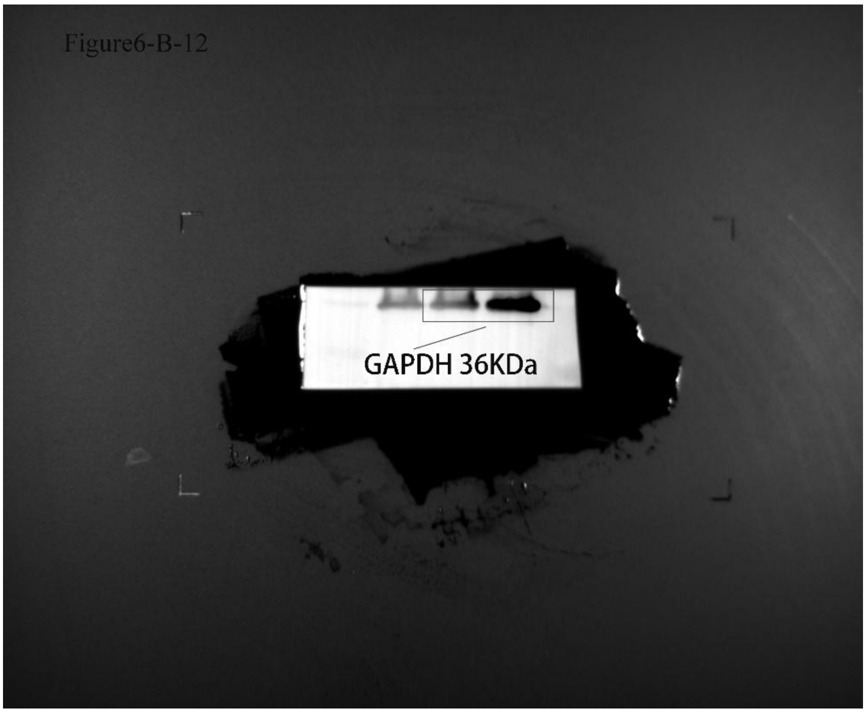

Figure6-E-1

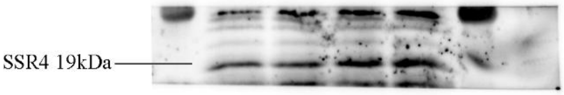

Figure6-E-2

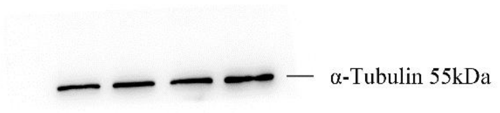

Figure6-E-3

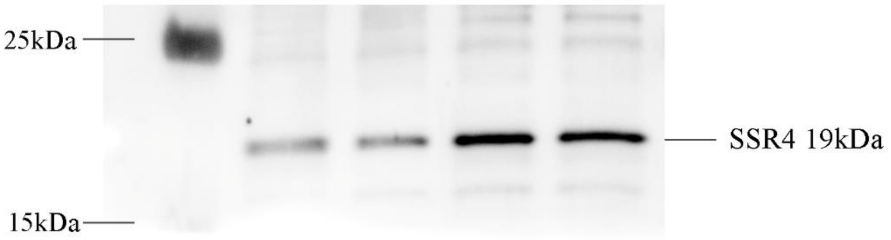

Figure6-E-4

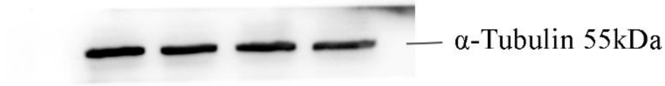

Figure6-E-5

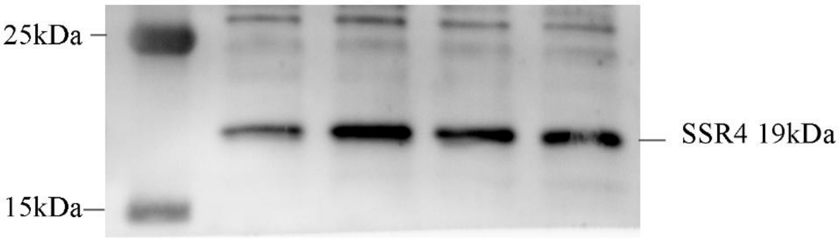

Figure6-E-6

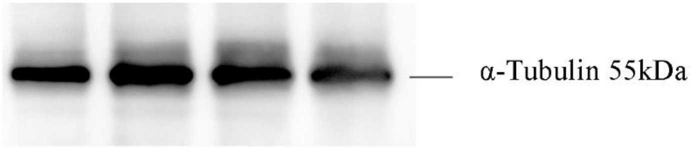

Figure6-G-1

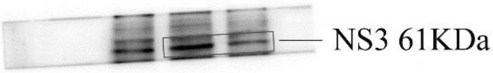

Figure6-G-2

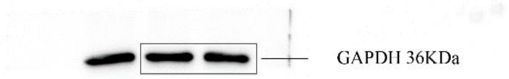

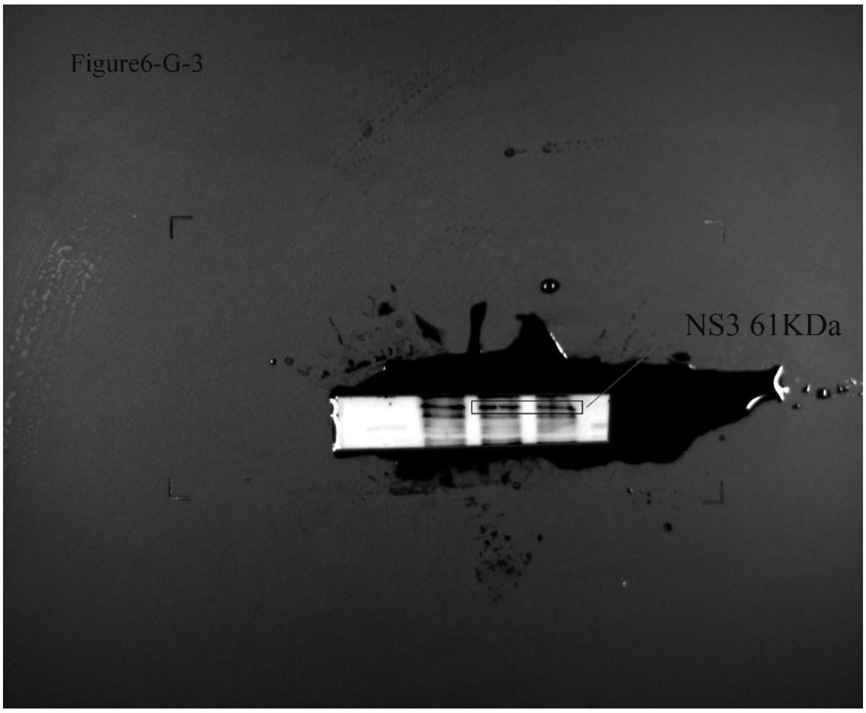

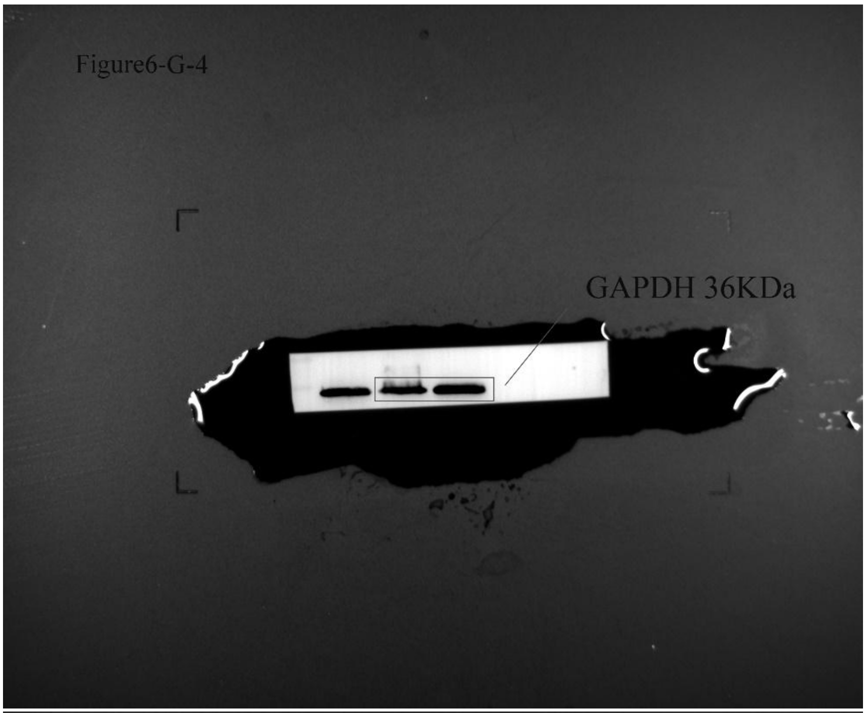
Figure6-G-4
GAPDH 36KDa

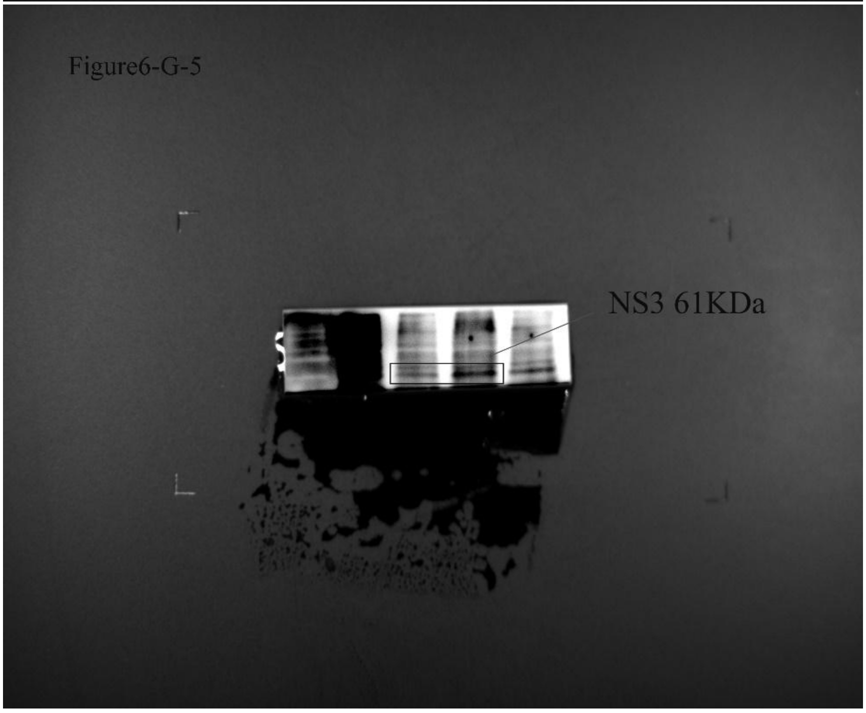
Figure6-G-5
NS3 61KDa

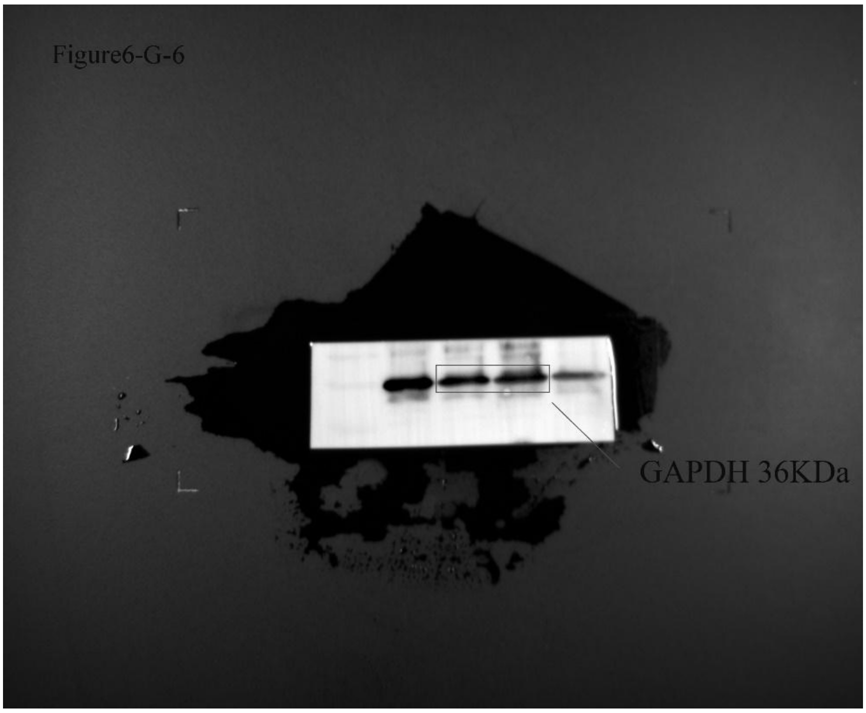
Figure6-G-6
GAPDH 36KDa
